# Novel magnetic properties of CoTe nanorods and diversified CoTe_2_ nanostructures obtained at different NaOH concentrations

**DOI:** 10.1080/14686996.2017.1317218

**Published:** 2017-05-15

**Authors:** Yu-Xi Lei, Nan-Xi Miao, Jian-Ping Zhou, Qadeer Ul Hassan, Jing-Zhou Wang

**Affiliations:** ^a^ School of Physics & Information Technology, Shaanxi Normal University, Xi’an, PR China; ^b^ School of Electrical & Information Engineering, North Minzu University, Yinchuan, PR China

**Keywords:** CoTe_2_, nanorods, nanostructures, magnetic properties, first-principles, 40 Optical, magnetic and electronic device materials, 102 Porous/Nanoporous/Nanostructured materials, 401 1st principle calculations, 203 Magnetics/Spintronics/Superconductors, 301 Chemical syntheses/processing

## Abstract

CoTe and CoTe_2_ nanorods with average diameter of 100 nm were synthesized by a simple hydrothermal process, and different CoTe_2_ nanostructures were obtained by changing the NaOH concentration. CoTe nanorods exhibit weak ferromagnetism while CoTe_2_ nanorods present paramagnetic behavior. Different magnetic behaviors occur in the other CoTe_2_ nanostructures due to Na^+^ entrance into CoTe_2_ crystals. A first-principles study on Na-doped CoTe_2_ confirms the magnetic characteristics.

## Graphical abstract

**Figure:**
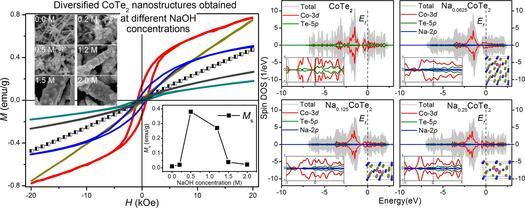

## 1. Introduction

Transition metal tellurides have been actively studied recently due to their unique properties with potential applications in optoelectronics [1,2], catalysis [3,4], electrode [5,6] and magnetism [7,8]. Tellurides with different morphologies and compositions presented different properties. The properties can be significantly enhanced in low-dimensional tellurides, which were manifested by experimental investigations and theoretical calculations [9,10]. Therefore, it is vital to fabricate tellurides with controllable morphology and selective composition by a facile synthesis strategy.

The crystal structure of cobalt telluride was determined in previous studies. CoTe has a NiAs type structure while CoTe_2_ exhibits a stable marcasite-type structure also called mattagamite [11–13]. The bulk CoTe_*x*_, obtained by a direct reaction method at high temperature and specific pressure, presented a ferromagnetic character in CoTe and a paramagnetic behavior when *x* was above 1.2 [14,15]. On the other hand, reports about nano CoTe and CoTe_2_ were limited in morphology, such as nanowires [16], nanorods [17], nanotubes [18,19] and other nanostructures [20]. A preparation of highly pure CoTe and CoTe_2_ nanocrystals with various morphologies and investigation on their magnetism in both experiment and theoretical calculation are comparatively lacking.

In this research, a simple hydrothermal process was used to synthesize CoTe and CoTe_2_ nanocrystals [21]. Uniform CoTe and CoTe_2_ nanorods were obtained by using an appropriate amount of ascorbic acid. Other CoTe_2_ nanostructures with different morphologies were also obtained by changing the NaOH concentration without any surfactant. The magnetic properties of the as-prepared CoTe nanorods and CoTe_2_ nanocrystals were investigated. The results indicate that CoTe nanorods, CoTe_2_ nanorods and other CoTe_2_ nanostructures exhibit different magnetic characteristics. The possible mechanism for such a substantial difference in magnetism was discussed based on first-principles calculations.

## 2. Experimental section

### 2.1. Sample preparation

In a typical synthesis of CoTe_2_ nanorods, 5 mmol Co(NO_3_)_2_·6H_2_O (purity 99%) and 2.0 g ascorbic acid (purity 99%) were dissolved in 100 ml distilled water, and mixed with 10 mmol TeO_2_ (purity 99.99%) under stirring for 5 min. Then, 50 ml ethanolamine (purity 99%) was added into the above mixture under constant stirring for 10 min. The dark red solution was transferred into a 200 mL Hastelloy autoclave. The autoclave was heated at 180°C for 20 h and cooled down to room temperature naturally. The final product was collected and washed with deionized water and absolute ethanol for several times, and dried at 60°C. For CoTe nanorods, we only increased the Co(NO_3_)_2_·6H_2_O to 10 mmol and kept other procedures unchanged. The other CoTe_2_ nanostructures were prepared with different NaOH concentrations (0.2, 0.5, 1.2, 1.5 and 2.0) used by keeping other procedures same as those for CoTe_2_ nanorods.

### 2.2. Measurements

The crystal structures of the as-synthesized samples were analyzed by X-ray diffraction (XRD) using a X-ray diffractometer (Rigaku, D/Max2550, Rigaku Corporation, Japan) with Cu Kα radiation (*λ* = 1.5406 Å) in the 2*θ* range of 10–70° under 40 kV and 100 mA. The measurement was performed using a step mode with a step of 0.02° and dwell time of 3 s per step. The morphological information and crystalline characteristics of the samples were examined by a field emission scanning electron microscopy (FESEM, FEI, Nova NanoSEM 450, USA) and a field emission transmission electron microscopy (FETEM, FEI Tecnai G^2^F20 S-Twin). X-ray photoelectron spectroscopy (XPS) measurements were performed with an Axis Ultra spectrometer (Kratos Analytical Ltd, Japan) using Al Kα radiation. The magnetic properties of the as-obtained samples were identified by a vibrating sample magnetometer (VSM) based on a superconducting quantum interference device (Cryogen-Free Magnet System, Cryogenic Ltd, UK). Magnetic hysteresis loops of CoTe and CoTe_2_ nanorods were collected at 10, 100 and 300 K. The other CoTe_2_ nanocrystals *M*-*H* loops were measured at 10 K.

## 3. Computational method

The electronic properties were studied using the Vienna *ab initio* simulation package (VASP) [22] within the MedeA^®^ software environment [23]. The projector augmented-wave (PAW) method [24] was adopted to calculate the exchange-correlation energy. The calculation was carried out in terms of the generalized gradient approximation (GGA) with Perdew–Burke–Ernzerhof (PBE) functional [25] based on the density functional theory (DFT). Self-consistent determination of spin-polarization was performed by using plane-wave basis set, and the cut-off energy was chosen to be 350 eV. Defect calculations were implemented using 1 × 2 × 1, 2 × 2 × 1, 2 × 2 × 2 supercells and 5 × 2 × 3, 3 × 2 × 3, 3 × 2 × 2 Monkhorst-Pack k-point mesh [26]. The self-consistent-field (SCF) was taken to be 10^−5^ eV. The optimized atomic positions were determined by relaxation until the Hellmann-Feynman force on each atom was less than 0.02 eV/Å.

## 4. Results and discussion

### 4.1. Phase and structure

**Figure 1.**
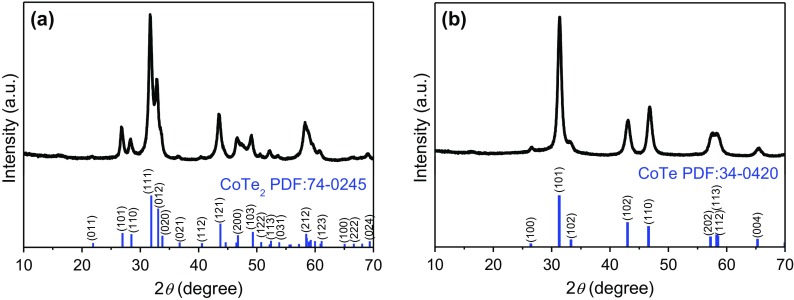
The XRD patterns of (a) CoTe_2_ nanorods synthesized at Te/Co molar ratio of 2 and (b) CoTe nanorods synthesized at Te/Co molar ratio of 1 in the absence of NaOH. The bottom stick patterns correspond to the standard CoTe_2_ PDF card (number 74-0245) and standard CoTe PDF card (number 34-0420).

Figure 1(a) shows the XRD pattern of the sample synthesized at Te/Co molar ratio of 2, with 2.0 g of ascorbic acid, and without NaOH; the pattern can be clearly indexed as orthorhombic mattagamite [PDF number 74-0245; space group: Pmnn(58); *a* = 3.882 Å, *b* = 5.301 Å and *c* = 6.298 Å]. This is consistent with previous reports [17,18]. No foreign peak was detected, suggesting a highly pure CoTe_2_ structure. However, the product was different when we change the molar ratio of tellurium dioxide and cobalt nitrate to 1:1 under same conditions, as shown in Figure 1(b). All of the reflections can be indexed to the hexagonal phase CoTe with NiAs structure [space group: P63/mmc (194), PDF number 34-0420; *a* = 3.892 Å and *c* = 5.374 Å], which is accordance with the other CoTe nanostructures [16,19]. No foreign peaks were detected, suggesting a highly pure CoTe. Thus, CoTe_2_ and CoTe product can be selectively synthesized by this approach.

**Figure 2.**
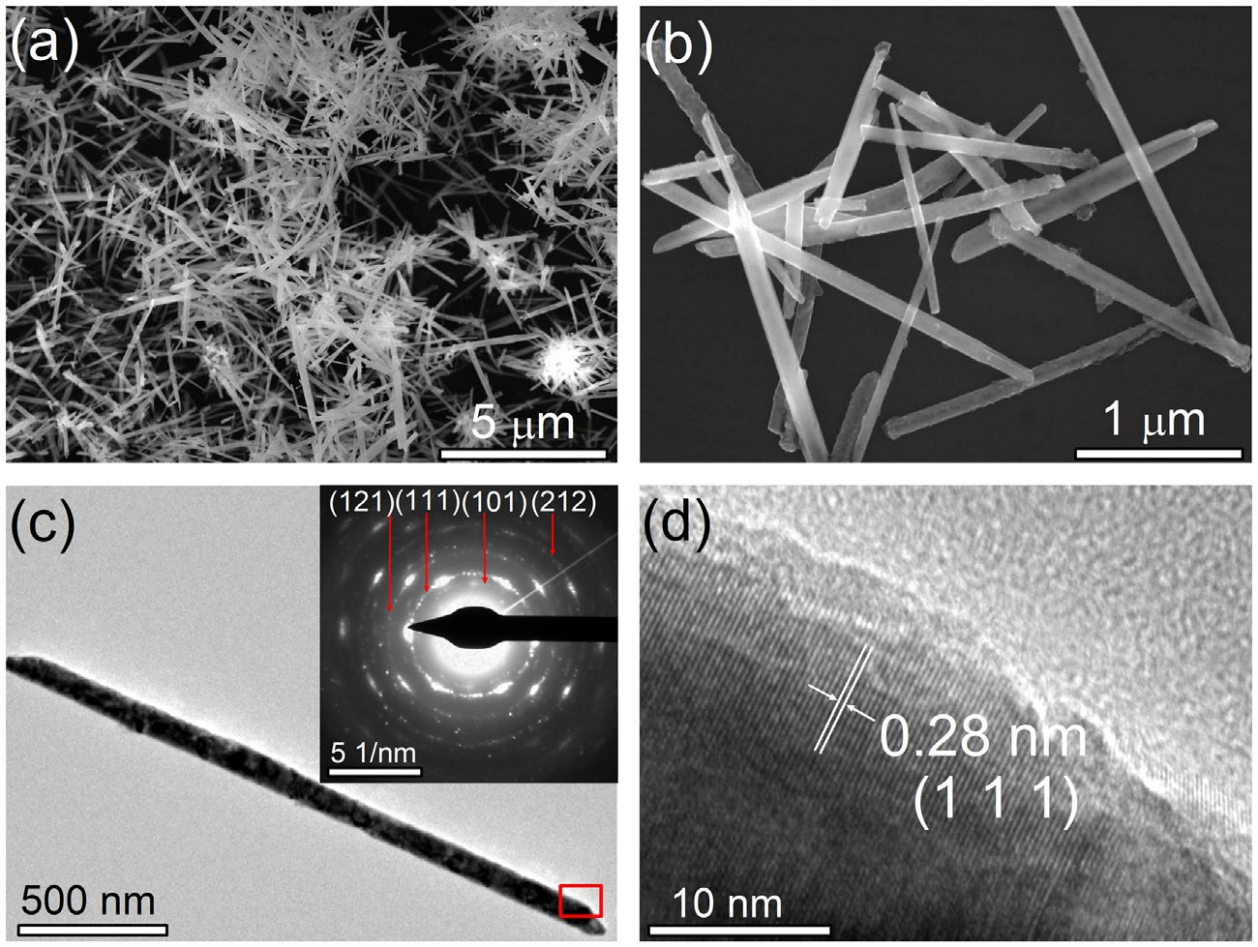
(a) Low and (b) high-resolution SEM images of the as-prepared CoTe_2_ nanorods synthesized at Te/Co molar ratio of 2 in the absence of NaOH. (c) Typical TEM image of an individual CoTe_2_ nanorod. The inset shows a corresponding SAED pattern; (d) HRTEM image taken from the red rectangular area in (c).

**Figure 3.**
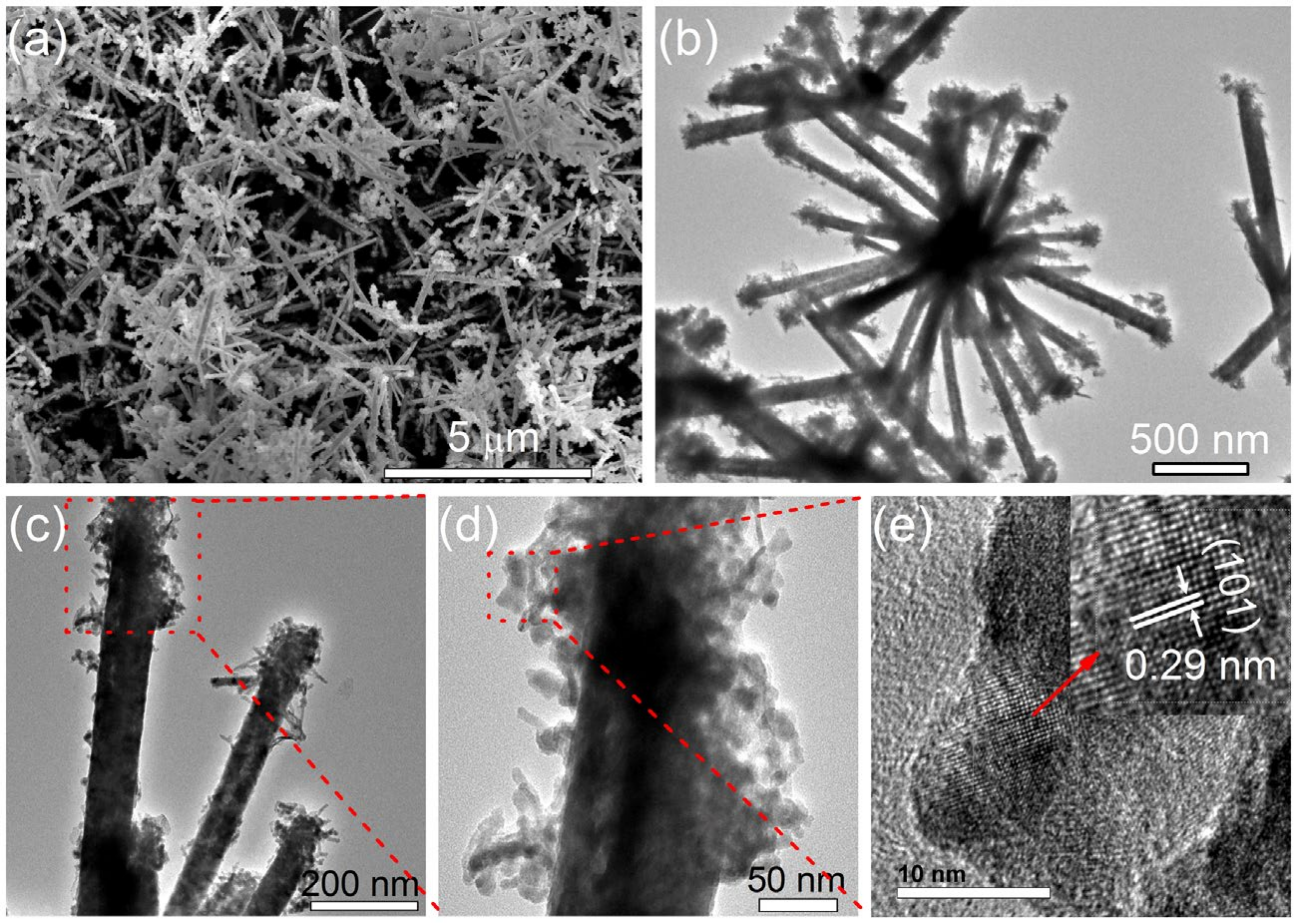
(a) Typical SEM image of the as-prepared CoTe nanorods synthesized at Te/Co molar ratio of 1 in the absence of NaOH. (b) Typical and (c, d) enlarged TEM images of CoTe nanorods; (e) HRTEM image taken from the red rectangular area in (d).

Figure 2(a) shows a typical SEM image of the as-prepared CoTe_2_ sample, revealing that a 1D nanostructure was achieved. The high-resolution SEM image in Figure 2(b) clearly shows that the CoTe_2_ nanostructures are uniform nanorods with about 100 nm in diameter and 2 μm in length. Figure 2(c) shows a representative TEM image of an individual CoTe_2_ nanorod, confirming that the nanorod has a long straight and solid structure with a sharp head. The inset shows a corresponding selected-area electron diffraction (SAED) pattern of the CoTe_2_ nanorod, where the polycrystalline diffraction rings can be indexed to the orthorhombic CoTe_2_. The discrete bright spots for the (1 1 1) planes indicate that the nanocrystals constitute a nanorod along with [1 1 1] crystallographic orientation, which is similar to CoTe_2_ nanotubes [18]. Figure 2(d) shows the high-resolution TEM (HRTEM) image in the red rectangle in Figure 2(c). The clear and uniform lattice fringe indicates the high crystallinity in the nanorod. The interplanar spacing 0.28 nm corresponds to (1 1 1) crystal plane in CoTe_2_. The morphological characteristics of the as-obtained CoTe in Figure 3 reveal nanorods with 100 nm in diameter and 1 μm in length. There is flocculation or thorns in the head of each nanorod. The observed interplanar spacing 0.29 nm corresponds to (1 0 1) crystal plane in CoTe.

**Figure 4.**
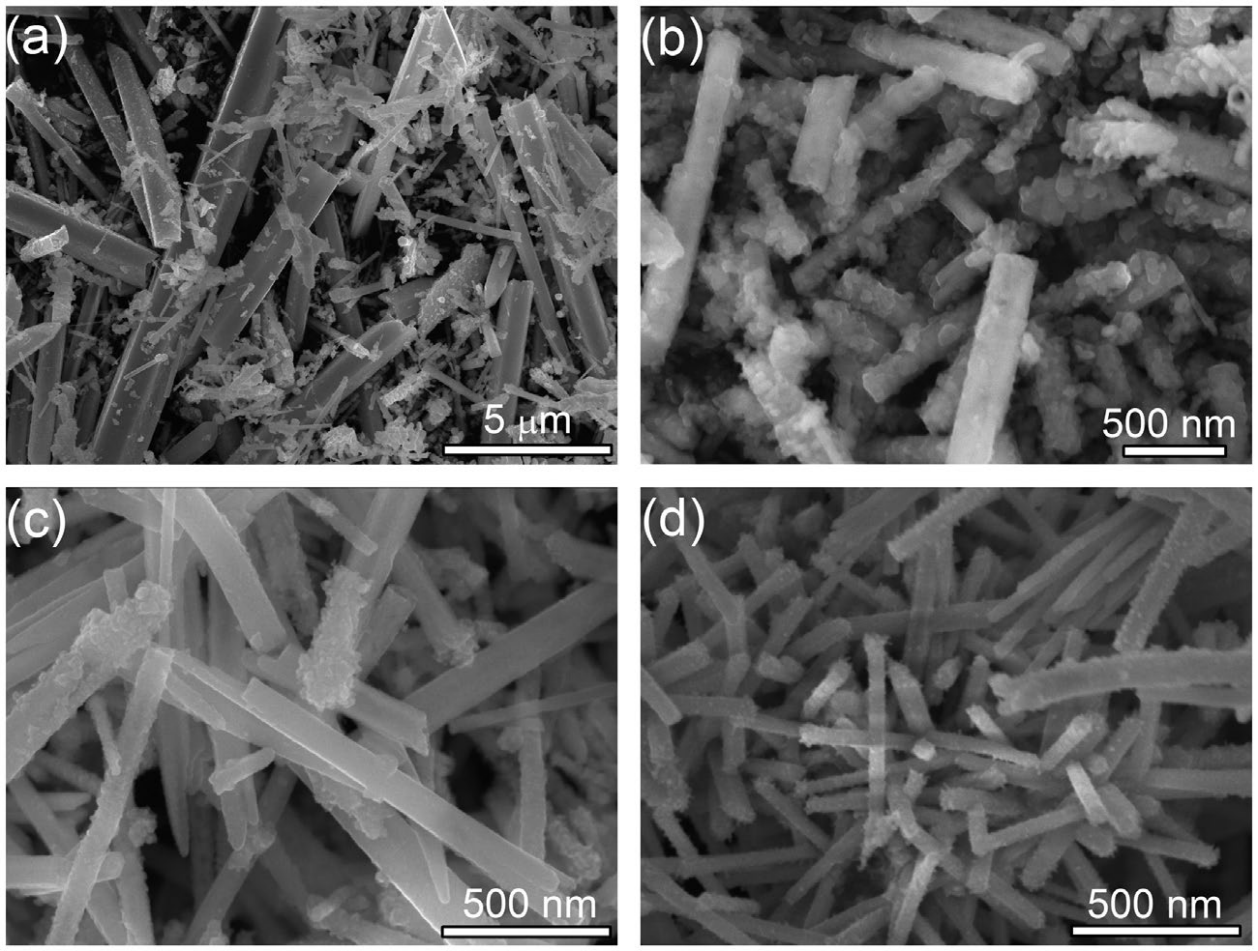
SEM images of CoTe_2_ products synthesized in the absence of NaOH at Te/Co molar ratio of 2 with (a) 0.0 g, (b) 0.5 g, (c) 3.0 g and (d) 5.0 g of ascorbic acid.

Ascorbic acid is a weak reducing agent [27] and would slowly reduce Te^4+^ to Te in solution at certain temperatures [28], playing a dominant factor in the formation of uniform CoTe_2_ nanorods. A series of experiments was conducted at different amounts of ascorbic acid, while keeping other experimental conditions the same. Non-uniform CoTe_2_ micro-rods and some irregular particles were obtained in the absence of ascorbic acid (Figure 4(a)). When a small quantity (0.5 g) of ascorbic acid was used, CoTe_2_ nanorods with a diameter about 200 nm (Figure 4(b)) were formed, but the nanorods are composed of numerous small particles with rough surfaces. When the amount of ascorbic acid was increased to 2.0 g, uniform CoTe_2_ nanorods with an average diameter of 100 nm were prepared as shown in Figure 2. The morphology of the products slightly changed when the amount of ascorbic acid was further increased, while the diameter of the as-prepared CoTe_2_ nanorods remained at ~100 nm as shown in Figure 4(c) and 4(d). Thus, the additive contributes to the formation of CoTe_2_ nanorods, and the optimal amount of ascorbic acid for the synthesis of uniform CoTe_2_ nanorods is above 2.0 g.

**Figure 5.**
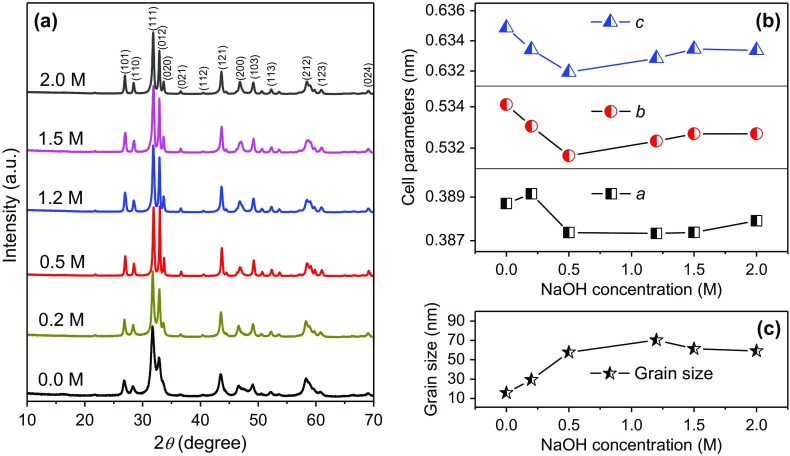
(a) XRD patterns, (b) lattice parameters and (c) grain size of CoTe_2_ samples synthesized at Te/Co molar ratio of 2 with different NaOH concentrations.

The alkali concentration plays a key role in hydrothermal process to influence the size and morphology of the products. Figure 5(a) shows the XRD patterns of samples with different NaOH concentrations, keeping the other conditions the same. All the samples share same orthorhombic CoTe_2_ phase. The relative intensity of (0 1 2) peak first increase then decrease with NaOH concentration. This means a preferred orientation occurs during crystal growth. Furthermore, there is a little change in cell parameters as shown in Figure 5(b), where the cell parameters were obtained by Rietveld refinement of XRD patterns using Maud software with an R-factor below 10. The mattagamite CoTe_2_ with space group Pmnn(58) (ICSD number 25678) was used as a basic model. In general, the lattice parameters decrease firstly with increasing NaOH concentration, reach minimum at 0.5 M and then gradually increase possibly due to Na entrance in CoTe_2_ crystal lattice. However, the difference in cell parameters is less than 0.004 nm, indicating negligible effect of NaOH concentration on the CoTe_2_ crystal structure. Figure 5(c) shows the fitted crystal size of CoTe_2_ samples with different NaOH concentrations. The crystal size obviously increases when NaOH concentration increases to 0.5 M, and remains unchanged with NaOH concentration.

**Figure 6.**
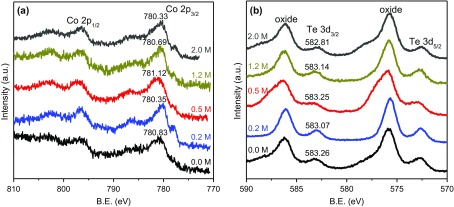
High-resolution XPS spectra of (a) Co 2p and (b) Te 3d for CoTe_2_ samples synthesized at Te/Co molar ratio of 2 with different NaOH concentrations.

XPS was employed to further investigate the Na entrance in crystal lattice. Figure 6(a) shows the high-resolution spectra of Co 2p region for CoTe_2_ nanostructures with different NaOH concentrations. The main peaks of Co 2p_3/2_ and Co 2p_1/2_ are located at about 781 eV and 796 eV, respectively, as expected for the cobalt telluride nanostructures [3,19]. Figure 6(b) shows the high-resolution spectra of the Te 3d region. The peaks at 573 eV and 583 eV correspond to Te 3d_5/2_ and Te 3d_3/2_. Peaks at 576 eV and 586 eV can be attributed to the oxidation of Te on the surface of products [29]. The binding energies of Co 2p and Te 3d decrease gradually with rising NaOH concentration, as shown in Figure 6. This means there are more electrons in the crystal of the product with high NaOH concentration, which mainly originates from entered Na in CoTe_2_ crystal. The binding energy for the sample with NaOH concentration of 0.5 M is higher than that of the sample without NaOH due to its smaller cell parameters. We could not detect Na peaks in XPS spectra because of the low Na content in the CoTe_2_ crystal.

**Figure 7.**
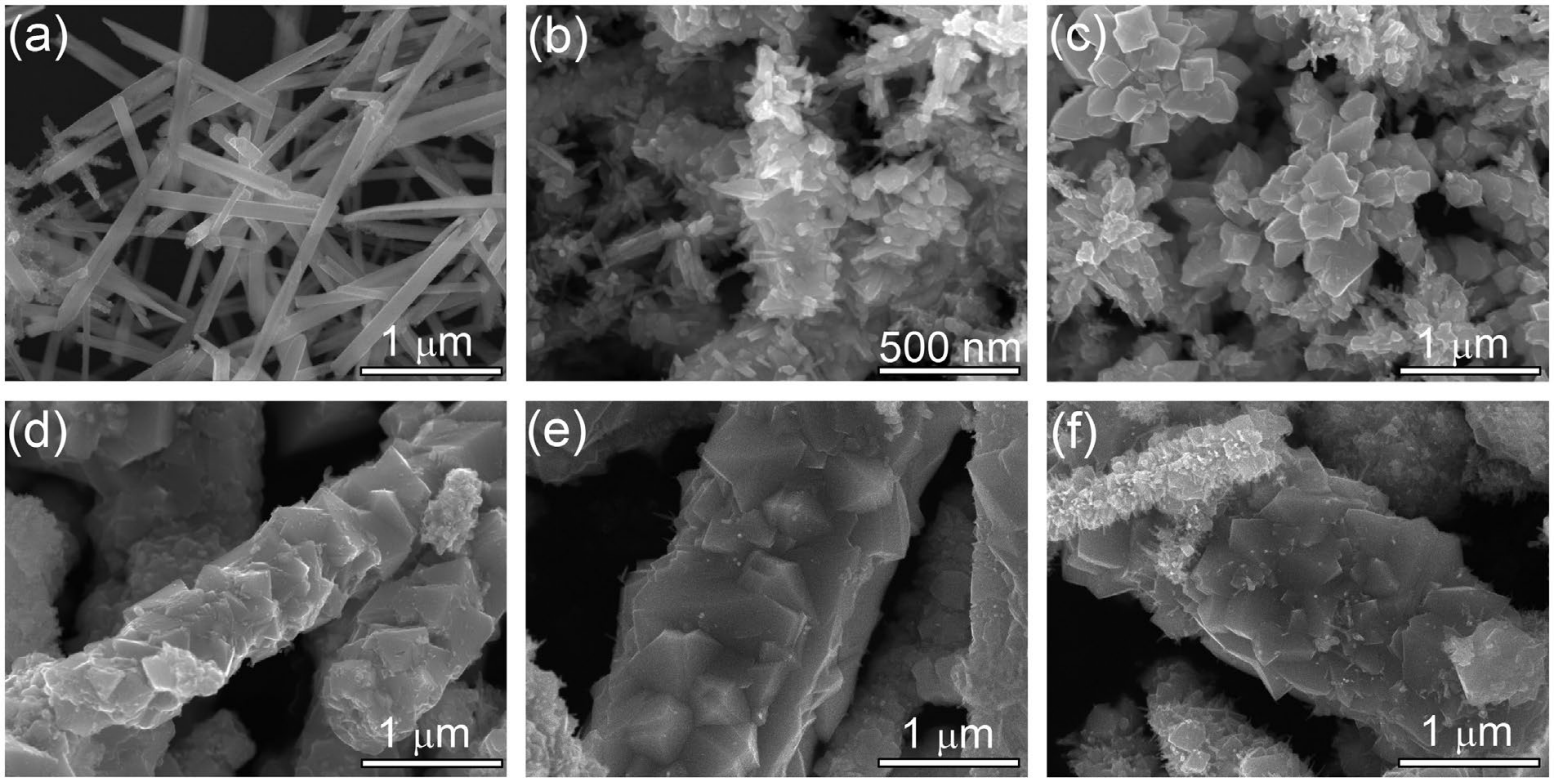
SEM images for CoTe_2_ samples synthesized at Te/Co molar ratio of 2 with different NaOH concentrations: (a) 0.0 M, (b) 0.2 M, (c) 0.5 M, (d) 1.2 M, (e) 1.5 M and (f) 2.0 M.

Typical SEM images of the samples with different NaOH concentrations are shown in Figure 7. Uniform CoTe_2_ nanorods (Figure 7(a)) can be achieved in the absence of NaOH. When NaOH concentration is 0.2 M, CoTe_2_ changes to short rods with numerous thorns (Figure 7(b)) from 1D nanorods. When NaOH concentration is 0.5 M, CoTe_2_ presents flower-like hierarchical structures (Figure 7(c)). When the NaOH concentration further increases to 1.2 M, a large rod composed of small cubes with diameter of about 1 μm and a rough surface was obtained (Figure 7(d)). The products show similar morphology when NaOH concentration further increases to 1.5 M and 2.0 M, both of which share microrod structure (Figure 7(e, f)) with that of 1.2 M. The trend in crystalline size observed from SEM agrees well with the fitting result (Figure 5(c)). We consider that the alkali concentration adjusts the reaction rate and reaction kinetics, and further affects the final morphology.

### 4.2. Magnetic properties

**Figure 8.**
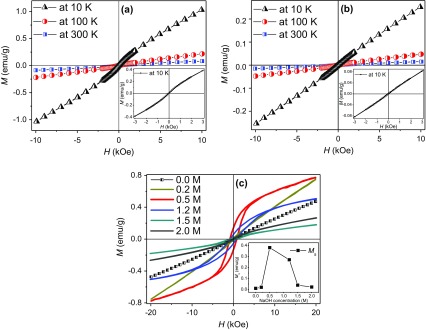
Hysteresis loops of (a) as-prepared CoTe nanorods and (b) CoTe_2_ nanorods measured at 10, 100 and 300 K. The insets display the blown-up loops measured at 10 K under low magnetic field. (c) Hysteresis loops of as-prepared CoTe_2_ samples with different NaOH concentrations measured at 10 K. The inset is the variation of saturation moment with NaOH concentration.


The hysteresis loops of as-prepared CoTe nanorods were measured at 10, 100 and 300 K, as shown in Figure 8(a). The inset is the enlarged loops at low magnetic field. The magnetic moment of CoTe nanorods is 0.08 emu g^–1^ under 10 kOe at 300 K, which is an order of magnitude less than the bulk CoTe value [14], possibly due to the size of CoTe nanorods. The moment increases to 0.21 and 1.03 emu g^–1^ under 10 kOe at 100 K and 10 K, respectively. There is a weak hysteresis in the *M*-*H* curves. This suggests that the pure CoTe nanorods exhibit a feeble ferromagnetic behavior, which is consistent with CoTe nanotubes [30], Figure 8(b) shows the hysteresis loops of CoTe_2_ nanorods. The inset is the enlarged hysteresis loops at low magnetic field. It can be seen that the magnetic moment of CoTe_2_ nanorods is 0.25 emu g^–1^ under 10 kOe at 10 K, and reduces to 0.047 and 0.015 emu g^–1^ at 100 and 300 K, respectively, which is less than those of CoTe nanorods. No clear obvious hysteresis is observed in the *M*-*H* curves. The results assure us that the pure CoTe_2_ nanorods exhibit a paramagnetic behavior and the magnetization decreases with the increasing temperature, which is in accordance with the classical paramagnetism theory (or Langevin paramagnetism) given by [31]:

(1)
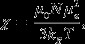



where *N* is the atomic number per unit volume.

Figure 8(c) shows the *M*-*H* curves of CoTe_2_ nanostructures with different NaOH concentrations at 10 K. The samples exhibit different magnetic characteristics. At low NaOH concentration (0.0 M and 0.2 M), the samples exhibit a paramagnetic feature. When NaOH concentration is 0.5 M, the sample manifests a ferromagnetic behavior with saturation moment (*M*_s_) of 0.38 emu g^–1^ and coercivity (*H*_c_) of 823 Oe. A line is fitted between the moment and magnetic field in the *M*-*H* curve at high magnetic field. The *M*_s_ is obtained by an intercept of the line on the *M* axis. A small decrease occurs in ferromagnetism with *M*_s_ of 0.27 emu g^–1^ when the NaOH concentration is 1.2 M. Then, *M*_s_ dramatically decreases to about zero in the 2.0 M sample. The inset in Figure 8(c) shows the variations of *M*_s_ with NaOH concentration, in which first a rise and then a drop are clearly illustrated. Generally, the magnetic properties of magnetic material directly relate to its size and shape [32]. Nevertheless, the variation in size of different CoTe_2_ nanostructures mainly occurs in the products with NaOH concentration between 0 M and others (Figures 5(c) and 7). In particular, the size is almost unchanged at NaOH concentrations above 0.5 M, unlike the trend of magnetic properties. The samples are obtained at same conditions except NaOH concentration, so we consider this magnetic difference originates mainly from the Na interaction in the CoTe_2_ crystal, which fill the holes of CoTe_2_ crystalline lattice and affect the electronic structure by hybridizing with Co and Te atoms.

### 4.3. First-principles study

**Figure 9.**
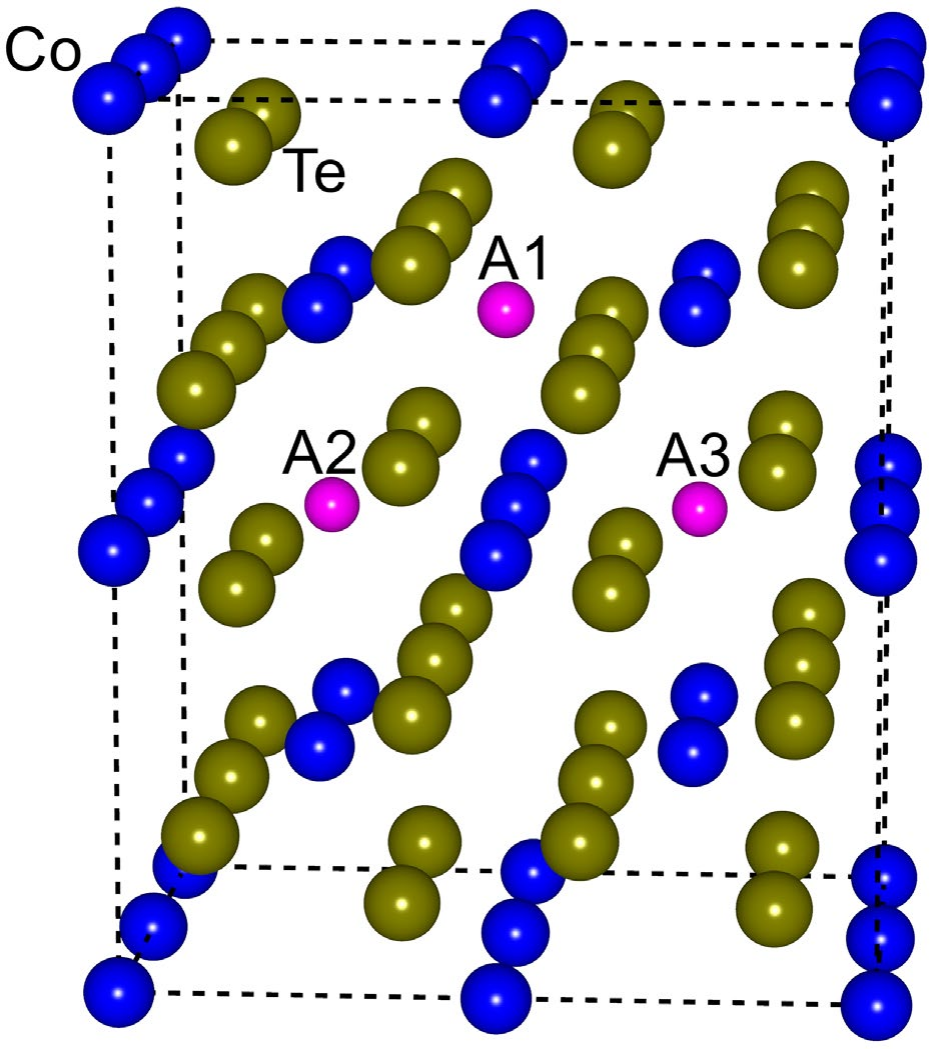
Three sites available for intercalated Na in a 2 × 2 × 2 supercell of CoTe_2_.


Six Te atoms are adjacent to Co atoms, forming a general ligand field in the mattagamite CoTe_2_ crystal, and each Co atom has a distorted octahedral space while each Te atom shares a distorted tetrahedral neighborhood [33]. Three distinct sites are available to accommodate intercalated sodium, marked as A1, A2, and A3 as shown in Figure 9. The formation energy of CoTe_2_ with an intrinsic defect is estimated based on:

(2)
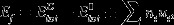



where 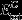 is the total energy of supercell including the intrinsic defect, 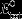 is the total energy of the host supercell excluding the defects, *n*_i_ is the number of atoms entered the host supercell to create an interstitial (−) or removed from the host supercell to create a vacancy (+), and *u*_i_ is the chemical potential of the corresponding atom [34]. We compared the formation energies of the Na-doped CoTe_2_ with different sites A1, A2, and A3. The A1 site has the least formation energy of 0.31 eV, meaning it is thermodynamically stable to form doped compounds.

**Figure 10.**
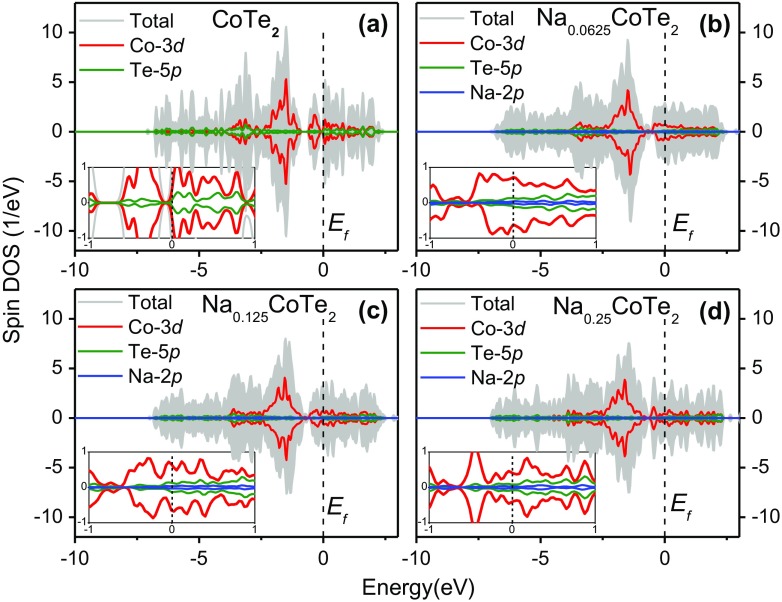
Spin-polarized total and main partial DOS for Na_*x*_CoTe_2_ with (a) *x* = 0, (b) *x* = 0.0625, (c) *x* = 0.125 and (d) *x* = 0.25. The inset is the blown-up view of DOS near the Fermi level. The black vertical dash lines at the zero energy give the Fermi energy levels.

Electronic properties were researched on these systems to determine their spin polarizations in the crystal. We calculated the density of states (DOS) of Na-doped Na_*x*_CoTe_2_ with a composition of *x* = 0, 0.0625, 0.125 and 0.25. The latter three models were performed by using Na doped with A1 site in 2 × 2 × 2, 2 × 2 × 1 and 1 × 2 × 1 supercells, respectively. It can be seen the DOS near the Fermi level is mainly from Co 3*d* and nearest-neighboring Te 5*p* states. Na 2*p* states have little contribution to the total DOS, as shown in Figure 10. There is a large exchange splitting around the Fermi level between the spin-up and spin-down bands for Co 3*d* states in Na-doped CoTe_2_, which provides the magnetic moment. The 3*d* electrons in transition metal atoms play a dominant role in the metallicity as well as the magnetic properties [35]. The small magnetic moment of Te originates from the hybridization of Co 3*d* states with nearest-neighboring Te 5*p* states [36]. Furthermore, the spin-polarization of Co 3*d* states at the Fermi level is 6.18, 8.14, 1.9, and 0.36% with the increase in Na concentration, indicating Na induces the electron transmission from 4*s* to 3*d* of Co. Moreover, the total DOS shows no energy gap near *E*_*f*_, indicating the metallic nature of the Na-doped CoTe_2_. With the increase in the Na concentration, the magnetic moment of the compound enhances at first, and then decreases gradually as shown in Table 1, in agreement with the experimental results.

**Table 1.** The calculated magnetic moments.

composition	*m* (*μ*_B_/unit)
CoTe_2_	0.0198
Na_0.0625_CoTe_2_	0.3532
Na_0.125_CoTe_2_	0.1909
Na_0.25_CoTe_2_	0.0068

## 5. Conclusions

In conclusion, uniform CoTe and CoTe_2_ nanorods with high purity and excellent crystallinity were synthesized via a facile hydrothermal route. CoTe_2_ nanostructures with diversified morphologies at different NaOH concentrations were also obtained using this approach. CoTe nanorods exhibit a weak ferromagnetism, while CoTe_2_ nanorods exhibit paramagnetic behavior. The other CoTe_2_ nanostructures present a first increase and then decrease in ferromagnetism with the increase in Na concentration. This phenomenon was explained on the basis of a first-principles study, which agreed well with experimental results. This research provides an effective route to synthesize telluride nanostructures and control the magnetism in Na-doped CoTe_2_.

## Disclosure statement

No potential conflict of interest was reported by the authors.

## Funding

This work was supported by the National Natural Science Foundation of China [grant number 51372148, 51672168]; Fundamental Research Funds of North Minzu University [grant number 2014XBZ02].
